# Utilizing Un-enhanced Chest Computed Tomography Screening for Blunt Trauma Surgery Decisions

**DOI:** 10.7759/cureus.69590

**Published:** 2024-09-17

**Authors:** Abdul Baseer, Nosheen Noor, Nasreen Aman, Ahmed Nasir Qureshi

**Affiliations:** 1 Cardiothoracic Surgery, Medical Teaching Institute, Lady Reading Hospital, Peshawar, PAK; 2 Radiology, Medical Teaching Institute, Lady Reading Hospital, Peshawar, PAK; 3 Surgery, Ghurki Trust Teaching Hospital, Lahore, PAK

**Keywords:** blunt chest trauma, chest radiograph, ct scan, hemothorax, lung contusion, pneumothorax

## Abstract

Background

Blunt chest trauma is a common and potentially life-threatening condition that requires prompt assessment for potential surgical intervention. Computed tomography (CT) of the chest has emerged as a valuable tool due to its heightened sensitivity and specificity in detecting thoracic injuries compared to conventional chest radiography.

Objective

This study aims to assess the impact of non-contrast CT chest findings on surgical decision-making and compare these findings with those from chest radiographs.

Methods

The study was conducted at the Accident and Emergency Department of Medical Teaching Institute, Lady Reading Hospital, Peshawar, Khyber Pakhtunkhwa, Pakistan. Patients of all ages and genders who presented with blunt chest trauma were included. Non-contrast CT chest scans were used as an initial screening tool and compared with traditional chest radiographs. Data collected included patient demographics, mechanism of injury, diagnostic findings, and treatment decisions. Imaging was performed using a GE Optima 16-slice scanner (Medsystems Sp. z o.o., Lublin, Poland).

Results

The study included 246 patients, of whom 210 (85.4%) were males. The most common age group was 50 years or older, comprising 71 (28.9%) of the sample. The predominant mechanism of trauma was road traffic accidents, reported by 188 (76.4%) patients. Hemopneumothorax was detected in 121 (49.2%) patients on CT scans compared to 34 (13.8%) patients on chest radiographs. On chest radiograph, the pneumothorax component was missed in 43 (17.5%) patients, and the hemothorax component was not detected in 21 (8.5%) patients. Patient management included conservative management in 30 (12.2%) cases and surgical intervention in the form of unilateral tube thoracostomy in 173 (70.3%) patients or bilateral tube thoracostomy in 43 (17.5%) patients.

Conclusion

Our study supports the use of non-contrast CT scans as a reliable diagnostic tool for blunt chest trauma, consistent with current literature. This approach facilitates prompt management decisions, particularly for initiating tube thoracostomy based on findings of pneumothorax and hemothorax. The rarity of mediastinal great vessel trauma further justifies minimizing routine contrast use, thereby enhancing the efficiency of trauma evaluations.

## Introduction

Blunt chest trauma, resulting from incidents such as motor vehicle collisions, falls, or physical assaults, is a significant contributor to morbidity and mortality, necessitating prompt and accurate evaluation in emergency settings to identify injuries requiring surgical intervention [1-4]. Conventional diagnostic methods, including chest radiographs and physical examinations, may fail to detect critical injuries, leading to potential treatment delays. In contrast, the utilization of CT chest scans has emerged as a superior diagnostic tool due to its heightened sensitivity and specificity in detecting thoracic injuries [5]. Non-contrast CT scans, particularly in emergency scenarios, offer numerous benefits such as rapid imaging, reduced risk of nephrotoxicity and allergic reactions, lower costs, and decreased radiation exposure compared to contrast-enhanced CT scans [5]. Moreover, non-contrast CT scans provide a reliable alternative to other imaging modalities like chest radiographs or ultrasound, with the added capability of detecting mediastinal injuries, including hematomas or tears, which may not be visible through other techniques [1,2,6,7]. This capability significantly enhances the timely decision-making process for healthcare professionals regarding patient care and treatment [1,2,6-8]. However, while non-contrast CT scans offer substantial advantages in emergency settings, it is important to recognize that they may lack the detailed visualization provided by contrast-enhanced CT scans, particularly for vascular injury assessment [9]. In such cases, a contrast-enhanced CT scan may be essential, requiring healthcare providers to carefully evaluate the patient's clinical condition to determine the most suitable imaging modality [9,10].

Rationale

Non-contrast CT scans are crucial in emergencies involving blunt thoracic trauma, where time is of the essence. The use of contrast agents can delay patient care and pose risks such as allergic reactions and nephrotoxicity. Non-contrast CT scans provide a cost-effective alternative with reduced radiation exposure. Additionally, no similar studies have been conducted in our region, making this research essential for generating region-specific data and insights that can enhance clinical practice and patient outcomes locally.

## Materials and methods

Study design and duration

This retrospective cross-sectional analysis was conducted over two years, from May 2022 to April 2024, in the thoracic surgery and radiology departments of Lady Reading Hospital, Medical Teaching Institute, Peshawar.

Sampling technique and sample size

A non-probability convenient sampling method was employed to select the study population. The sample size was calculated using the OpenEpi calculator, with a minimum of 246 patients included to achieve a 95% confidence interval.

Inclusion and exclusion criteria

The study included patients admitted to the thoracic surgery department with blunt chest trauma who underwent non-contrast CT chest scans in the accident and emergency department, while patients with penetrating chest trauma were excluded.

Data collection

After obtaining research and ethical approval from the Institutional Review Board (IRB) (257/LRH/MTI; Dated March 11, 2024), retrospective data of blunt chest trauma patients admitted to the thoracic surgery department between May 2022 and April 2024 were retrieved from the hospital information management system (HIMS). Non-contrast CT chest scans were performed in the emergency department using the GE Optima 16-slice scanner (Medsystems Sp. z o.o., Lublin, Poland), and the images were evaluated on the picture archiving and communication system (PACS) system by a consultant radiologist who was a fellow of the College of Physicians and Surgeons Pakistan. The management of these patients was carried out by thoracic surgery consultants and residents, and the outcomes were extracted from the PACS system.

The collected data included patient demographics, mechanisms of injury, diagnostic findings, and treatment decisions. Only patients who underwent a CT chest scan were included in the study, while those who had only a chest radiograph without a subsequent CT scan were excluded. Additionally, patients referred from other hospitals who brought hard copies of their radiographs directly proceeded to a CT scan, and since their radiographs were not available in our HIMS, only the CT findings were recorded.

Statistical analysis

Frequencies and percentages, were used to summarize categorical variables. Chi-square test was used to assess the correlation between CT findings and patient management decisions, with a p-value of less than 0.05 demonstrating a significant association between imaging results and management choices. Statistical analyses were conducted using SPSS (IBM Corp. Released 2020. IBM SPSS Statistics for Windows, Version 27.0. Armonk, NY: IBM Corp).

## Results

The study included 246 patients, of whom 210 (85.4%) were males, with the most common age group being 51 years or more, constituting 71 (28.9%) of the sample (Table 1). The most prevalent mechanisms of trauma were road traffic accidents, reported by 188 (76.4%) patients, and falls, which accounted for 56 (22.8%) cases (Table 2).

**Table 1:** Patient Age

Age Group	N (%)	Cumulative Percentage (%)
10 years or less	25 (10.2%)	10.2
11 to 20 years	31 (12.6%)	22.8
21 to 30 years	24 (9.8%)	32.5
31 to 40 years	43 (17.5%)	50.0
41 to 50 years	52 (21.1%)	71.1
51 years or more	71 (28.9%)	100.0
Total	246 (100.0%)	100.0

**Table 2:** Mechanism of Blunt Injury

Mechanism of Injury	N (%)	Cumulative Percentage (%)
History of fall	56 (22.8%)	22.8
Mine collapse	1 (0.4%)	23.2
Physical assault	1 (0.4%)	23.6
Road traffic accident	188 (76.4%)	100.0
Total	246 (100.0%)	100.0

The most frequent injuries identified included rib fractures, hemothorax, pulmonary contusion, pneumothorax, and hemopneumothorax. Rib fractures were detected on radiographs in 127 (51.6%) patients, while CT scans identified fractures in 194 (78.9%) patients, with displaced fractures being the most common type, seen in 155 (63.0%) patients (Table 3).

**Table 3:** Rib Fracture Type on CT Scan

Rib Fracture Type	N (%)	Cumulative Percentage (%)
Displaced	155 (63.0%)	63.0
Undisplaced	25 (10.2%)	73.2
Comminuted	14 (5.7%)	78.9
Mixed	52 (21.1%)	100.0
Total	246 (100.0%)	100.0

Chest radiographs demonstrated a sensitivity of approximately 80% and a specificity of 97.7%. Among other fractures, clavicular fractures were detected in 28 (11.4%) patients on CT scan compared to 25 (10.2%) on the radiograph. In comparison, scapular fractures were present in 39 (15.9%) patients on CT scan and 19 (7.7%) on the radiograph. Sternal fractures were found in 9 (3.7%) patients on CT scan, vertebral body fractures in 8 (3.3%) patients, and transverse process fractures in 4 (1.6%) patients; none of these fractures were diagnosed on plain radiographs.

Hemopneumothorax was detected in 121 (49.2%) patients on CT scans, while chest radiographs identified this condition in only 34 (13.8%) patients. The pneumothorax component was missed in 43 (17.5%) patients on chest radiographs, and the hemothorax component was undetected in 21 (8.5%) cases.

Isolated pneumothorax was present in 28 (11.4%) patients on CT scan and hemothorax in 29 (11.8%) patients. Interestingly, more isolated pneumothorax and hemothorax cases were reported on the radiograph - 38 (15.4%) and 62 (25.2%) cases respectively, likely due to the missed diagnosis of hydropneumothorax on the radiograph.

Pneumomediastinum was present in 31 (12.6%) patients on CT scan compared to 11 (4.5%) patients on the radiograph, while mediastinal hematoma was found in only 1 (0.4%) patient on CT scan and was not detected on the radiograph. Lung contusions were observed in 148 (60.2%) patients on CT scans, whereas only 60 (24.4%) patients showed lung contusions on the radiograph. Additionally, two (0.8%) patients had manubriosternal joint dislocation identified on CT scan, none of which were detected on chest radiographs.

Patient management strategies included conservative management in 30 (12.2%) patients, unilateral tube thoracostomy in 173 (70.3%) patients, and bilateral tube thoracostomy in 43 (17.5%) patients (Table 4).

**Table 4:** Management Type

Management Type	N (%)	Cumulative Percentage (%)
Unilateral tube thoracostomy	173 (70.3%)	70.3
Conservative	30 (12.2%)	82.5
Bilateral tube thoracostomy	43 (17.5%)	100.0
Total	246 (100.0%)	100.0

Table 5 shows that hemopneumothorax identified on CT scans led to 87 (71.9%) cases receiving unilateral tube thoracostomy and 29 (24.0%) receiving bilateral tube thoracostomy. Pneumothorax management included 8 (28.6%) conservative treatments, 12 (42.9%) unilateral tube thoracostomies, and 8 (28.6%) bilateral tube thoracostomies. Hemothorax resulted in 3 (10.3%) conservative treatments, 21 (72.4%) unilateral tube thoracostomies, and 5 (17.2%) bilateral tube thoracostomies. Rib fractures were mostly managed with unilateral tube thoracostomy in 122 (91.0%), with only 2 (1.5%) requiring bilateral tube thoracostomy. Other injuries were managed conservatively in 4 (25.0%) cases, with unilateral tube thoracostomy in 11 (68.8%) and bilateral tube thoracostomy in 1 (6.2%) case (Table 5).

**Table 5:** Management Based on Imaging Findings

Imaging Findings	Conservative Management	Unilateral Tube Thoracostomy	Bilateral Tube Thoracostomy	Total	p-value
Hemopneumothorax (CT)	5 (4.1%)	87 (71.9%)	29 (24.0%)	121	0.008
Pneumothorax (CT)	8 (28.6%)	12 (42.9%)	8 (28.6%)	28	0.04
Hemothorax (CT)	3 (10.3%)	21 (72.4%)	5 (17.2%)	29	0.017
Rib Fractures (CT)	10 (7.5%)	122 (91.0%)	2 (1.5%)	134	0.026
Other Injuries (CT)	4 (25.0%)	11 (68.8%)	1 (6.2%)	16	0.047
Total	30	173	43	246	-

.

## Discussion

Our analysis of 246 blunt chest trauma cases revealed an increasing incidence with age, with 25 (10.2%) cases occurring in children under 10 years and 71 (28.9%) cases in individuals aged 51 years or older. Sikander et al. reported that the elderly population (>60 years) had the highest incidence, accounting for over 25% of cases [11]. Another study found an incidence of over 35% in individuals over 65 years, which aligns with our observations [12]. Additionally, Minervini et al. noted a 5-12% incidence in children, consistent with our finding of 25 (10.2%) [13].

Road traffic accidents (RTAs) emerged as the leading cause of blunt chest trauma, with 188 (76.4%) cases, followed by falls, which accounted for 56 (22.8%) cases. This finding aligns with previous studies that identified RTAs as the primary cause, representing 40-60% of cases [14,15]. Falls were notably prevalent among the elderly, accounting for 20-30% of cases [12,14].

Males constituted 210 (85.4%) of cases, consistent with the findings of Khursheed et al., who reported 82.5% male involvement. Other studies have similarly highlighted the higher prevalence of blunt chest trauma among males, often attributed to their increased participation in high-risk activities [16,17].

CT scans demonstrated greater accuracy than radiographs in detecting rib fractures, revealing 194 (78.9%) cases compared to 127 (51.6%) cases identified on radiographs (Table 6).

**Table 6:** A Comparison of Findings Detected on X-ray and CT Scan *Shows significant difference in a row

Injury Type	X-ray Findings (N (%))	CT Scan Findings (N (%))	p-value
Rib fractures	127 (51.6%)	194 (78.9%)*	0.001
Clavicular fractures	25 (10.2%)	28 (11.4%)	0.654
Scapular fractures	19 (7.7%)	39 (15.9%)*	0.014
Sternal fractures	0 (0%)	9 (3.7%)*	0.002
Vertebral fractures	0 (0%)	8 (3.3%)*	0.003
Transverse process fractures	0 (0%)	4 (1.6%)*	0.041
Hemopneumothorax	34 (13.8%)	121 (49.2*%)	0.001
Pneumothorax	38 (15.4%)	28 (11.4%)	0.285
Hemothorax	62 (25.2%)	29 (11.8%)*	0.001
Pneumomediastinum	11 (4.5%)	31 (12.6%)*	0.008
Mediastinal hematoma	0 (0%)	1 (0.4%)	0.312
Lung contusions	60 (24.4%)	148 (60.2%)*	0.001
Manubriosternal joint dislocation	0 (0%)	2 (0.8%)	0.218

Given this, we recommend CT scans when radiograph results are inconclusive, supported by Karul et al., who found CT scans to have higher sensitivity and specificity for detecting rib fractures [18]. Similarly, Parreira et al. advocated for CT scans in cases where rib fractures are suspected but not confirmed by radiographs [19].

A chi-square test revealed a significant association between CT scan findings and radiographs (p < 0.001), despite limitations due to low expected counts. Comparable findings were reported by Pagoti et al. and Livingston et al., confirming the superior accuracy of CT scans [20,21].

Regarding hemothorax, radiographs showed a sensitivity of 97.6% and a specificity of 87.9%. Tataroglu et al. reported 62.5% sensitivity and 100% specificity. For pneumothorax, radiograph sensitivity was 59.6% with a specificity of 91.8%, consistent with Tataroglu et al., who noted 39.1% sensitivity and 100% specificity [22].

The superior diagnostic performance of CT scans underscores their importance for accurately diagnosing hemothorax, pneumothorax, and hemopneumothorax. A significant association (p < 0.05) was found between CT scan findings and management approaches, with most cases (173 or 70.3%) receiving unilateral tube thoracostomy. Kim et al. also reported a significant correlation between CT-detected thoracic injuries and management decisions [23].

CT scans led to more aggressive management strategies, including bilateral tube thoracostomy in 43 (17.5%) cases, compared to only 27 (11.0%) for radiographs. This suggests that CT scans' diagnostic accuracy results in more aggressive management strategies. Similar studies support this finding.

Lung contusion was observed in 148 (60.1%) patients on CT scan and in 60 (24.4%) patients on chest radiograph. The sensitivity of radiographs in diagnosing lung contusion is 31.0%, indicating that radiographs miss a significant number of lung contusion cases that would be detected by CT scans. Lewis et al. also reported lung contusion as a common finding in blunt chest trauma (70%), with CT being more sensitive than radiographs in diagnosing early cases [24]. Suboptimal radiograph image quality, often due to the inability to perform standard posteroanterior projections, contributes to low detection rates, as discussed by several scholars [25,26].

This retrospective study evaluated non-contrast CT scans for blunt chest trauma, revealing that all but one patient with a non-expanding mediastinal hematoma were managed without contrast-enhanced CT. Other studies have underscored the efficacy of non-contrast CT scans as an initial screening tool for identifying patients requiring urgent surgical intervention and timely management [1,2,4,5,10]. Naidoo et al. confirmed that mediastinal vessel injuries are less common in blunt thoracic trauma [27].

Conducted within the emergency department of a high-volume tertiary care facility, this study highlights the efficacy of non-contrast CT scans as an initial screening tool for blunt chest trauma. Given the high capacity of the emergency radiology unit, the initial use of unenhanced CT scans for all patients deviates from the standard contrast-enhanced CT protocol, with post-contrast scans performed only if deemed necessary.

Study limitations

This study has several limitations. First, the retrospective nature may introduce selection bias, as only patients with available CT and radiograph data were included. Additionally, the study was conducted in a single tertiary care center, which may limit the generalizability of the findings. The use of non-probability convenient sampling may also affect the representativeness of the sample. Finally, the study did not compare outcomes of patients who underwent contrast-enhanced CT scans, which could provide a more comprehensive assessment of the diagnostic accuracy of non-contrast CT scans.

## Conclusions

Our study's findings are consistent with current literature advocating for the use of non-contrast-enhanced CT scans as a reliable diagnostic tool in blunt chest trauma. This approach facilitates accurate and prompt management decisions, particularly in initiating tube thoracostomy based on initial imaging findings of pneumothorax and/or hemothorax. The rarity of mediastinal great vessel trauma further supports minimizing routine contrast administration, thereby enhancing the efficiency and timeliness of trauma evaluations.

Non-contrast CT scans offer a cost-effective, low-risk alternative to contrast-enhanced imaging, with reduced potential for allergic reactions and nephrotoxicity. The ability to rapidly assess and accurately diagnose critical thoracic injuries using non-contrast CT scans ensures that patients receive timely and appropriate care. Implementing non-contrast CT as a standard practice in trauma settings could lead to more streamlined protocols and improved patient outcomes. Overall, our findings highlight the importance of adopting non-contrast CT scans in emergency departments to optimize diagnostic accuracy and expedite management in blunt chest trauma cases.
